# Bacterial Community Profile of the Gut Microbiota Differs between Hypercholesterolemic Subjects and Controls

**DOI:** 10.1155/2017/8127814

**Published:** 2017-06-15

**Authors:** Camilo Rebolledo, Alejandro Cuevas, Tomás Zambrano, Jacquelinne J. Acuña, Milko A. Jorquera, Kathleen Saavedra, Claudia Martínez, Fernando Lanas, Pamela Serón, Luis A. Salazar, Nicolás Saavedra

**Affiliations:** ^1^Center of Molecular Biology and Pharmacogenetics, Department of Basic Sciences, Faculty of Medicine, Universidad de La Frontera, Temuco, Chile; ^2^Department of Preclinical Sciences, Faculty of Medicine, Universidad de La Frontera, Temuco, Chile; ^3^Applied Microbial Ecology Laboratory, Department of Chemical Sciences and Natural Resources, Scientific and Technological Bioresource Nucleus, Universidad de La Frontera, Temuco, Chile; ^4^Faculty of Health, Universidad Santo Tomás, Temuco, Chile

## Abstract

The role of gut microbiota in the development of metabolic illnesses has been abundantly demonstrated. Recent studies suggest that gut microbiota alterations may also be related to the development of hypercholesterolemia. Therefore, we aimed to assess differences in the gut bacterial community profiles between hypercholesterolemic subjects and controls. Thirty cases diagnosed with hypercholesterolemia and 27 normocholesterolemic controls were included. A fasting whole blood sample was obtained to determine the lipid profile. In parallel, stool samples were collected and total DNA was isolated to assess the bacterial community profiles by denaturing gradient gel electrophoresis (DGGE). In addition, the Richness, Shannon-Weaver, and Simpson indexes were used to evaluate the richness and diversity of bacterial communities. As expected, serum concentrations of total cholesterol, triglycerides, and LDL-cholesterol were significantly higher in the cases compared with controls. Moreover, DGGE analysis showed a lower richness and diversity of bacterial communities in hypercholesterolemic subjects. In conclusion, our results showed differences in the profiles of bacterial communities between hypercholesterolemic subjects and controls, suggesting a possible role of the gut microbiota in the development of hypercholesterolemia.

## 1. Introduction

Cardiovascular diseases (CVD) are the leading cause of death in the world, causing annually about 16 million deaths and more than 21,000 in Chile [1, 2]. Projections are not promising, since it is expected that by 2030 these numbers should increase [3]. Among CVD, cardiac and cerebral ischemic diseases correspond to the most frequent presentations, having as a common etiologic mechanism the development of atherosclerosis in arteries irrigating the compromised tissues [4]. On the other hand, dyslipidemias are considered among the most important risk factors contributing to CVD development. Considering that hypercholesterolemia, defined by the elevation of circulating low-density lipoprotein levels, increases the risk of coronary artery disease, its treatment—besides reducing cholesterol levels—has been shown to decrease both incidence and mortality by acute coronary events [5, 6].

During the last years, gut microbiota has been related to both the maintenance of health and the development of several diseases [7]. The role of microbiota in disease development has been strongly demonstrated in the case of inflammatory bowel diseases (IBD) such as Crohn's disease and ulcerative colitis [8, 9]. In this context, changes in the bacterial profiles have been also associated with metabolic disturbances such as obesity and type 2 diabetes mellitus [10, 11]. Recent studies have suggested the influence of changes in the microbiota on lipid metabolism in animal models, describing differences in both cholesterol absorption and serum levels between germ-free and conventional rats [12, 13]. Moreover, studies performed in hamsters—animals showing human-like lipoprotein metabolism [14]—have shown that specific bacterial groups found in stool samples are related to improvements in lipid metabolism induced by diet changes [15].

Nowadays, precision medicine has sought to improve the understanding of the health-disease process, where a broader description of factors involved in the development of a particular pathology becomes highly relevant. The prior statement led to suggesting that gut microbiota characterization represents a useful aspect into a better comprehension of the cardiovascular health status of an individual [16]. Considering these antecedents, the aim of the present study was to assess differences in the gut bacterial community profiles between hypercholesterolemic subjects and controls.

## 2. Materials and Methods 

### 2.1. Subjects

We design a case-control study including 30 hypercholesterolemic subjects (cases) diagnosed according to the NCEP criteria [17] and 27 normocholesterolemic subjects (controls), selected at the Center for Cardiovascular and Internal Medicine Studies, Universidad de La Frontera (Temuco, Chile). Anthropometric parameters such as systolic blood pressure (SBP) and diastolic blood pressure (DBP), weight, and height were measured. Individuals diagnosed with IBD, diabetes mellitus, and obesity were excluded considering that these pathologies have been associated with changes in the composition of the gut microbiota. Individuals with history of antibiotic therapy during the prior 6 months were also excluded. The present study was conducted based on the Declaration of Helsinki, and all individuals agreed voluntarily to participate by signing a written informed consent, previously approved by the Scientific Ethics Committee of Universidad de La Frontera (number 030/2015).

### 2.2. Biochemical Determinations

Biochemical measurements were performed on serum samples obtained from total blood using standard venipuncture techniques following a 12-h overnight fast. Glucose, triglycerides (TG), total cholesterol (TC), and high-density lipoprotein cholesterol (HDL-C) were quantified using enzymatic-colorimetric methods. Low-density lipoprotein cholesterol (LDL-C) was calculated using Friedewald's formula when TG did not exceed 4.52 mmol/L. Quality of the determinations was controlled by using normal and pathological commercial serums (Human, Germany).

### 2.3. Molecular Analysis

Stool samples were collected to analyze bacterial community profiles of the gut microbiota by gel electrophoresis with denaturing gradient (DGGE) technique. For the DGGE, DNA was isolated from deposition samples using the Fast DNA Spin (MP Biomedicals, USA) commercial kit, following the manufacturer's instructions. DNA was later quantified by UV spectrophotometry with Infinite® 200 PRO Nanoquant microplate reader (Tecan, Switzerland). Then, 100 ng DNA was used to amplify a 454 bp fragment of the 16S rRNA gene by touchdown PCR using the EUBf933-GC and EUBr1387 primers [18].

PCR was performed in 50 *μ*L of final volume containing MgCl2 (3 mM), GoTaq® Flexi Buffer (1x), dNTPs (0.2 mM each), GoTaq FlexiDNA polymerase (1.25 units), and primers (200 nM each). Touchdown PCR was performed on a MultiGene™ thermocycler (Labnet International, Inc., USA) using the following cycling conditions: denaturation at 94°C for 1 minute, followed by a hybridization step using a 0.5°C decrease for each cycle between 65 and 55°C, with 10 additional cycles at 55°C, and the extension step at 72°C for 3 minutes. A final extension of 7 minutes was included. PCR products were visualized by electrophoresis on a 2% agarose gel using a 100 bp DNA marker stained with GelRed™ (Biotium, USA).

Subsequently, PCR products were loaded onto a DGGE gel (acrylamide/bisacrylamide in 37.5 : 1 ratio) at 8% (w/v) in 1x TAE buffer (20 mM acetic acid and 1 mM EDTA pH 8.0). A gradient of 40 to 60% of denaturing agents (urea 7 M and 40% formamide) was used in DGGE gel preparation. Electrophoretic separation was performed at 20 V for the initial 10 minutes and then at 80 V for 17 hours at constant 60°C. Finally, DGGE gels were stained using SYBR® Gold (Invitrogen™) and the images were acquired using a Geldoc-It® TS2 310 photodocumentation system (UVP, USA). Banding patterns were analyzed in the CLIQs 1D Pro software (Totallab Ltd., UK). A nonmetric multidimensional scaling (nMDS) data matrix incorporating the Bray-Curtis similarity index was obtained from the Primer6 software (Primer-E Ltd., UK). In addition, we analyzed the pattern of bands to evaluate richness and diversity by the Shannon-Weaver and Simpson indexes.

### 2.4. Statistical Analysis

Statistical analysis was performed using the IBM SPSS Statics software for Mac, version 23.0 (IBM Corp., USA). Data are presented as averages ± standard deviation (SD). Differences between means of continuous variables were evaluated by Student's* t*-test. Statistical significance was set at *p* < 0.05.

## 3. Results

Anthropometric and biochemical variables of the subjects are summarized in Table 1. Analyzed individuals were aged between 40 and 80 years, with no significant differences between the two groups. The results did not show significant differences for SBP and DBP parameters. As expected, serum concentrations of TC and LDL-C were significantly higher in cases (6.13 and 3.99 mmol/L, resp.) compared with controls (5.36 and 3.01 mmol/L, resp.). These individuals also showed higher elevated glucose and TG levels (5.70 and 1.91 mmol/L, resp.) compared with controls (5.22 and 1.18 mmol/L, resp.). However, no significant differences were identified for HDL-C levels. Cases also showed significantly higher BMI (24 Kg/m^2^) than controls (23 Kg/m^2^).

**Figure 1:**
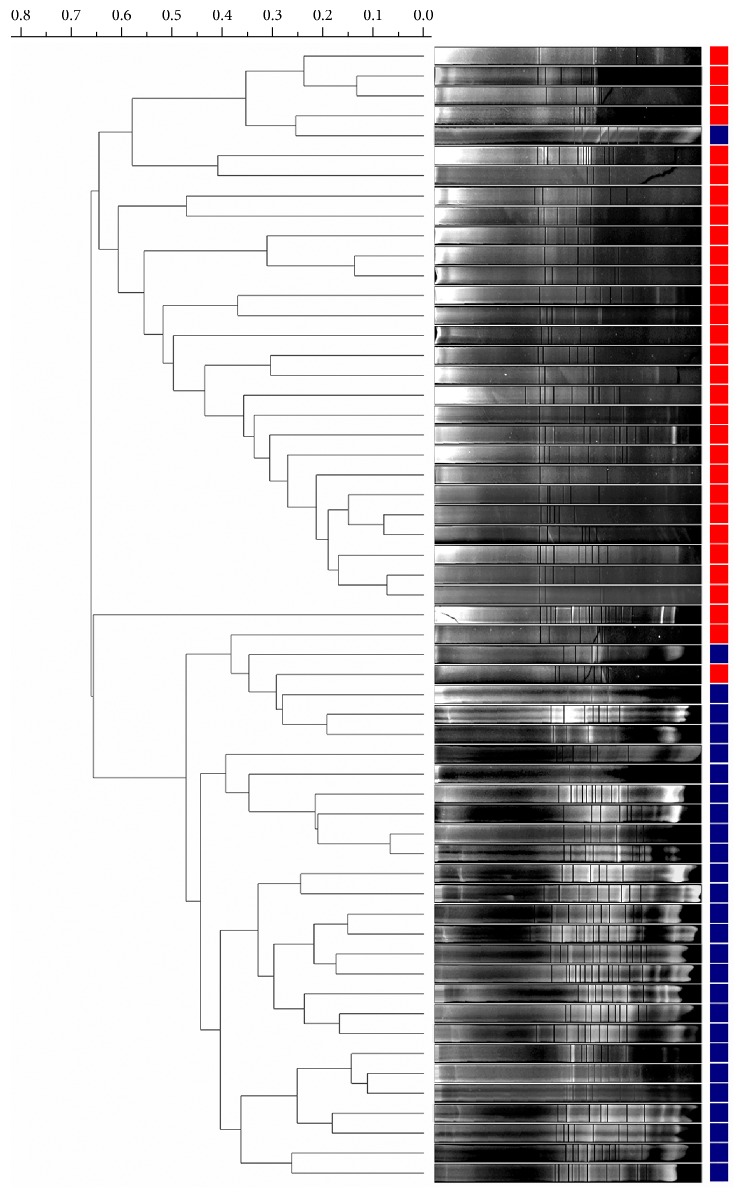
Dendrogram of bacterial community profiles in stool samples from hypercholesterolemic subjects (red) and controls (blue).

With respect to the molecular analysis performed by DGGE, we observed that the banding profiles differ between cases and controls, which in general were grouped in the two main branches in the dendrogram (Figure 1).

**Figure 2:**
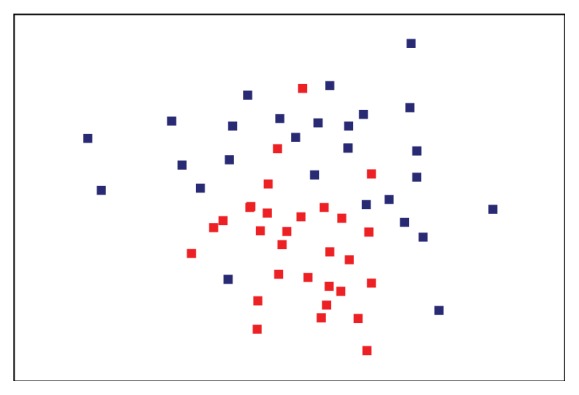
nMDS analysis of bacterial community profiles in stool samples from hypercholesterolemic subjects (red) and controls (blue).

Then, this difference was also confirmed by plotting the nMDS analysis, which shows that cases are similar to each other, forming a well-defined grouping (Figure 2).

Finally, we found significant differences for the Richness, Shannon-Weaver, and Simpson indexes of the banding profiles between both groups, with a noticeable decrease of the three indexes used in cases with respect to the controls (Table 2).

## 4. Discussion

A very well established association exists between hypercholesterolemia and CVD risk. However, up until now, the wide spectrum of CVD determinants is yet far to be fully understood. Very recently, a clear association between variations in gut microbiota and disease development has been demonstrated, showing a growing interest in the relationship between gut microbes and lipid profile. In the present study, we explored possible differences between bacterial community profiles of the gut tract of hypercholesterolemic individuals and controls. By calculating diversity indexes obtained from the analysis of bacterial DNA from the gut, we have consistently demonstrated that hypercholesterolemic subjects have significantly lower bacterial diversity compared to control individuals. In addition, the nMDS scaling analysis demonstrated the existence of differences among cases and controls, allowing the grouping by similarity of 26 from the 29 hypercholesterolemic individuals.

Data obtained are concordant with a 2015 cross-sectional study relating microbiota with BMI, TG, and HDL-C, revealing that healthy lipid levels were associated with an increase in microbial diversity [19]. Moreover, a recent and highly relevant review provided insight into the relationship between the gut microbiota and interindividual variation in the lipid profile, suggesting that gut microbes can explain a significant part of these differences [20]. The calculated indexes, which account for bacterial diversity, reflected that significant differences exist in the number of phylotypes observed, also showing differences in the relative abundance of a taxonomic unit between the studied groups.

An interesting result of our study is that hypercholesterolemic individuals presented lower richness and lower diversity compared with control individuals. It has been suggested that attenuation in phylotypes number and alterations in bacterial relative abundance as showed by the studied hypercholesterolemic subjects could modulate cholesterol absorption or modulate cholesterol metabolism at the systemic level. In line with this observation, it has been demonstrated that facultative and anaerobic bacteria located in the large bowel produce secondary bile acids from bile salts secreted by the individual, which can modulate lipid metabolism at both hepatic and systemic levels [21]. On the other hand, the attenuation of certain communities of gut microorganisms could determine a lower production of short chain fatty acids (SCFAs), which have been shown to reduce plasma concentrations of cholesterol in rodents and humans [22], or they could mediate L-carnitine metabolism into forming trimethylamine N-oxide, a compound involved in the reverse transport of cholesterol, cholesterol and sterols metabolism, and the modulation of bile acids amount and composition [23].

The approach used in the present study does not permit to identify the specific communities that differ between the groups. However, since it has been suggested that differences in microbial diversity could directly affect hepatic metabolism by exerting an influence on hepatic lipid biosynthesis [24] as well as lipids degradation [25] due to its proximity to the gastrointestinal tract, differences described in the present study might affect this mechanism depending on the bacterial communities differing between hypercholesterolemic and control individuals. In this line, Claus et al. demonstrated that gut microbiota stimulates an increase in hepatic triglyceride synthesis and modulates essential lipid absorption regulators, such as taurocholate and tauromuricholate, associating this effect to the Coriobacteriaceae family [26]. Gut microbiota is also able to modify lipid fractions in serum, adipose tissue, and liver [27]. For example, Erysipelotrichi (Firmicutes phylum) has been associated with both lipid impairment in animal models of hypercholesterolemia and with the development of fatty liver in humans [28, 29].

Although the microbiota in hypercholesterolemic individuals has not been characterized, a possible role of bacteria as lipid profile modulators in humans has been reported in individuals whose diet was supplemented with probiotics, which are defined as live microorganisms administered in suitable amounts to confer health benefits on the host. Two randomized, double-blind, placebo-controlled studies showed a 4.4% TC and 6.2% LDL-C reduction caused by dairy products enriched with* Lactobacillus acidophilus* or* Enterococcus faecium*, in subjects having a normal lipid profile and subjects with medium to moderate hypercholesterolemia [30, 31]. In addition, the lipid-lowering effect of* Lactobacillus reuteri* was described as an effect associated with increased intraluminal bile acid deconjugation, resulting in reduced absorption of sterols different than cholesterol [32].

Among the limitations of our study, we need to mention that DGGE allows describing qualitatively the bacterial populations present in a particular sample. However, as aforementioned, our approach does not permit to identify which microorganisms could be differentially contained in the group of hypercholesterolemic subjects. Despite this, from a more general perspective, the nMDS ordination plot of the DGGE data showed a clear grouping of the microbiota from hypercholesterolemic individuals, which is not so evident in the control group, with the exception of some of these individuals that appear overlapping with the control group, suggesting that there is a group of predominant phylotypes influencing hypercholesterolemia.

Finally, the technical limitations could be corrected in future studies by analyzing microorganisms by means of next-generation sequencing (NGS) methodologies, allowing a much deeper description of the bacterial populations associated with the development of hypercholesterolemia.

## 5. Conclusion

Our results showed differences in the profiles and diversity of bacterial communities between hypercholesterolemic subjects and controls, suggesting a possible role of the gut microbiota in the development of hypercholesterolemia.

## Figures and Tables

**Table 1:** Clinical and demographic characteristics of hypercholesterolemic and controls subjects.

Parameter	Cases	Controls	*p* value
Age, years	64.0 ± 1.9	60.1 ± 2.2	0.100
SBP, mm Hg	127.1 ± 3.3	122.0 ± 4.1	0.328
DBP, mm Hg	81.2 ± 1.7	80.0 ± 2.3	0.655
BMI, Kg/m^2^	24.2 ± 0.4	22.9 ± 0.4	0.023
Glucose, mmol/L	5.70 ± 0.13	5.22 ± 0.12	0.008
TC, mmol/L	6.13 ± 0.30	5.36 ± 0.16	0.029
LDL-C, mmol/L	3.99 ± 0.27	3.01 ± 0.12	0.002
HDL-C, mmol/L	1.62 ± 0.09	1.60 ± 0.06	0.840
TG, mmol/L	1.91 ± 1.18	1.18 ± 0.09	0.001

SBP: systolic blood pressure; DBP: diastolic blood pressure; BMI: body mass index; TC: total cholesterol; LDL-C: low-density lipoprotein cholesterol; HDL-C: high-density lipoprotein cholesterol; TG: triglycerides.

**Table 2:** Shannon-Weaver, Simpson, and Richness indexes of bacterial community profiles from hypercholesterolemic subjects and controls.

Index	Cases	Controls	*p* value
Shannon-Weaver	1.80 ± 0.43	2.07 ± 0.43	0.019
Simpson	6.23 ± 2,92	7.02 ± 2.62	0.025
Richness	7.10 ± 3.24	9.41 ± 3.15	0.009
